# Treating schistosomiasis among South African high school pupils in an endemic area, a qualitative study

**DOI:** 10.1186/s12879-018-3102-0

**Published:** 2018-05-25

**Authors:** Andrea Lothe, Nqobile Zulu, Arne Olav Øyhus, Eyrun Floerecke Kjetland, Myra Taylor

**Affiliations:** 1 Norwegian Centre for Imported and Tropical Diseases, Department of Infectious Diseases Ullevaal, Oslo University Hospital, 0424 Oslo, Norway; 2 Institute for Global Development and Planning, University of Agder, 4630 Kristiansand, Norway; 3 Centre for Communication, Media and Society, University of KwaZulu-Natal, Durban, 4001 South Africa; 4 Discipline of Public Health Medicine, Howard College Campus, University of KwaZulu-Natal, Room 219, George Campbell Building, Science Drive, Durban, 4001 South Africa

**Keywords:** Schistosomiasis, Mass-Treatment Campaign, Pupils, South Africa, Health Belief Model

## Abstract

**Background:**

Schistosomiasis, a neglected tropical disease caused by parasites that infest open water sources such as rivers and dams may increase susceptibility to HIV. Mass-treatment with praziquantel tablets, recommended by the World Health Organization reduces the prevalence of schistosomiasis. The goal in endemic areas is 75% treatment participation in every treatment round (e.g. yearly). However, in rural Ugu district, KwaZulu-Natal, South-Africa there was low participation among pupils in a Department of Health Mass-Treatment Campaign for schistosomiasis.

**Methods:**

Nested in a large study on schistosomiasis the study was conducted in 2012 over 4 months using qualitative methods with the Health Belief Model as the conceptual framework. Purposive sampling was done. Focus Group Discussions were undertaken at six schools in grades 10–12. Individual in-depth interviews were held with one teacher and two pupils at each school. In addition three traditional healers and a community health worker were interviewed.

**Results:**

The severity of schistosomiasis was not recognised and neither was the pupils’ susceptibility. Barriers to treatment included confusing *S, haematobium* symptoms with sexually transmitted infections, teasing and stigma.

**Conclusions:**

Increased knowledge, health literacy for treatment, and correct understanding about the severity of schistosomiasis may provide cues to action. The study indicates that comprehensive information may increase pupil participation in mass-treatment and decrease schistosomiasis prevalence.

**Trial registration:**

This study was registered with clinicaltrials.gov registry database and the registration number is NCT01154907 30 June 2011.

## Background and rationale

Schistosomiasis (Bilharzia) is the second most important disease in terms of public health impact after malaria, and people in 52 countries are at risk of the infection [1]. Schistosomiasis is an endemic, chronic and disabling disease [2]. Urogenital schistosomiasis (*Schistosoma (S.) haematobium*) may present itself as female genital disease and increase susceptibility to HIV [3–8]. Schistosomiasis is one of the worlds’ neglected tropical diseases, particularly affecting the poor in developing countries, especially in rural areas [9], and can lead to, or increase poverty through undernourishment, pain and anaemia [10]. Furthermore, there are deleterious effects on cognition, school performance and morbidity linked to schistosomiasis [10]. Several studies indicate that the damage from this disease may remain obscure (sub-clinical) or mimic sexually transmitted diseases and the consequences may last for decades in patients that have missed treatment when they were young [11–13]. The estimation is that 25% of the population in Sub-Saharan Africa may be infected with schistosomiasis [14]. In South Africa it has been estimated that 4.5 million people are infected with *S. haematobium* and there are some foci of *S. mansoni*, the majority of who live in KwaZulu-Natal (KZN), Eastern Cape, Limpopo, Gauteng, and Mpumalanga Provinces [15–17].

Schistosomiasis is managed by mass-treatment with praziquantel [16, 18, 19] used in many countries with the aim to control and finally eliminate the disease [20]. Praziquantel is the preferred drug of treatment [19]. It has been successfully used in mass-treatment campaigns in many countries endemic for schistosomiasis [21].

The advantages of praziquantel include that it kills the worm and the lack of severe side effects [19]. Depending on the local prevalence of infection the WHO strategy is to treat 75% of school aged children with praziquantel annually or every 2 years, depending on the prevalence of schistosomiasis, aiming at reducing the prevalence as well as schistosomiasis related morbidity [21]. Although treatment is provided in schools at no cost, participation in KwaZulu-Natal, South Africa has been variable and there is a need to identify factors that may influence a pupil’s participation in the mass-treatment campaign [22]. This study aims to explore attitudes towards anti-schistosomal treatment.

## Methods

### Setting of the study

The study was conducted in the Ugu District, KwaZulu-Natal, South Africa. The selected schools had all participated in the Department of Health (DoH) Mass-Treatment Campaign against schistosomiasis [22]. Schools were situated in rural areas (as defined by the Department of Education), and pupils came from traditional households of wattle and daub or more recent buildings using blocks or bricks.

### Study design

**Fig. 1:**
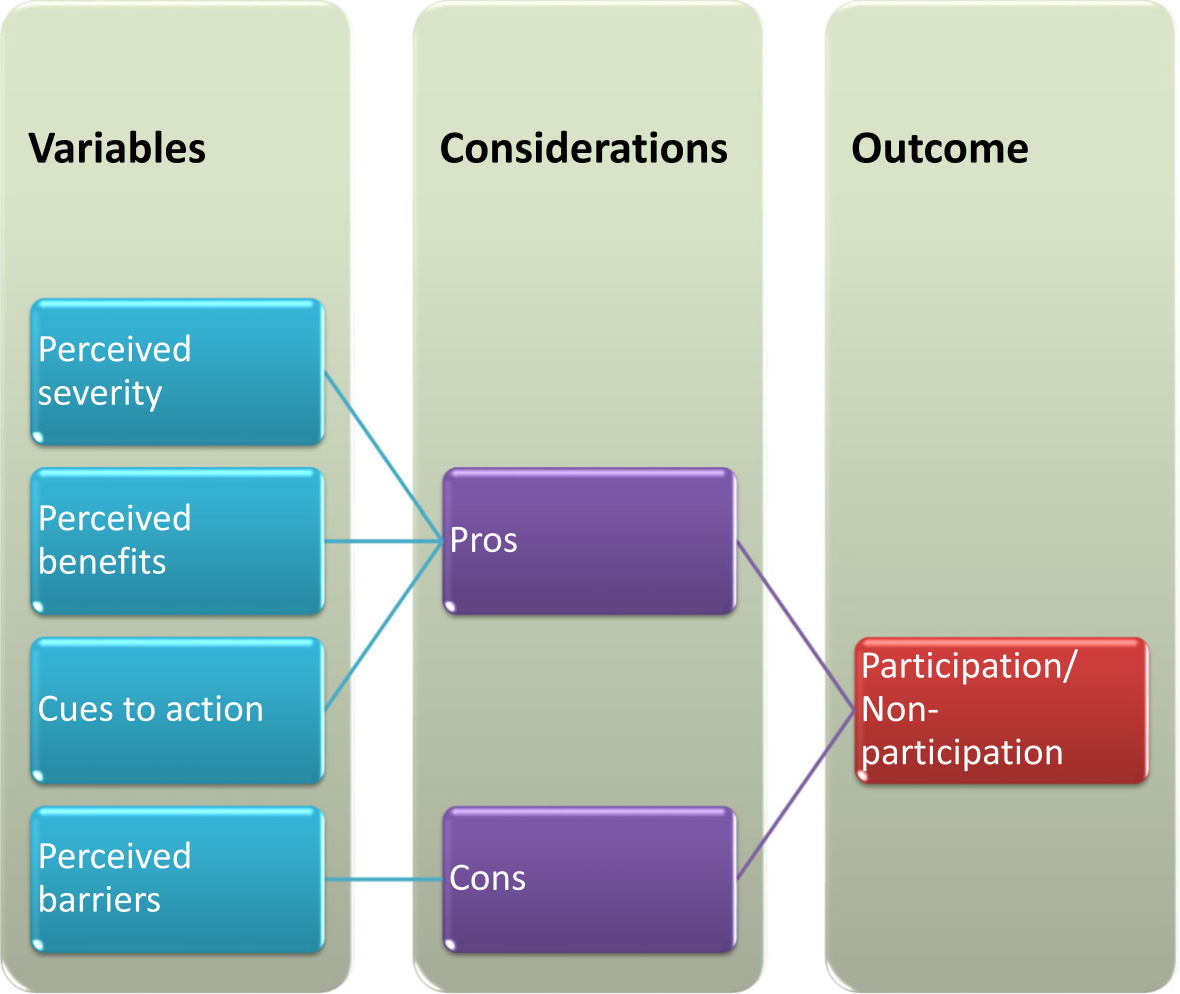
Rosenstock’s Health Belief Model Adapted from Rosenstock [23]

A combination of in-depth interviews, and focus group discussions (FGDs) were undertaken. Secondary data, from the Department of Health and previous research findings from the area, played an important part in the initial phase of the research. Local health professionals provided cultural advice and background information. The qualitative methods aimed for an in-depth understanding of the phenomena and employed a small study sample where percentages are not really meaningful. The Health Belief Model was used as the conceptual framework. It is based on the assumption that recipients will decide whether or not to accept treatment after considering the advantages and disadvantages [23]. Figure 1 shows the constructs influence the behaviour either in support of or against treatment. The extent to which this occurs determines whether or not a person will take the treatment.

### Characteristics of participants

This study used a qualitative method’s approach, selecting purposively pupils from 6 schools, 3 of which had high (50–75%) and 3 of which low (10–20%) participation in the DoH Mass-Treatment Campaign. There are 530 schools in Ugu but of these only 16 schools situated below 300 m altitude had been included in the DoH Mass-Treatment Campaign [22]. Schools have on average 400 pupils per school [22]. The pupils were numbered to indicate whether they attended schools with a high participation rate (A,B,C) or a low participation rate (D,E,F). The learner study participants were all purposively selected from pre-defined (different) sites in each class room, e.g. third row by the window and back row left corner, then next class in different pre-defined sites. Only one teacher per school who had not been assigned to help with the mass-drug administration was invited for an interview, One community health worker and three traditional healers were invited. They were identified by people living in the communities as healers who were trusted to help others with health issues.

### Processes and models

Each school was visited, the study was explained and the headmaster or his representative allocated a teacher to the project. Appointments were made for the researcher to conduct the interviews. Interview schedules were developed for the focus group discussions (FGD) and for the in-depth interviews and translated into isiZulu. These explored the respondents’ perceptions about schistosomiasis, causation, transmission, prevention and treatment and the anti-schistosomal mass-treatment campaign. The life cycle of schistosomiasis, schistosomiasis type, and quantifiable data were not explored in this study.

In each school two focus group discussions were held for boys and girls respectively with up to seven participants from grades 10–12 in each group. In addition at each school individual in-depth interviews were held with one female pupil, one male pupil (from grade 10–12) and one teacher.

The sessions (1–2 h’ duration) were recorded using a tape recorder placed in the middle of the group/near interviewee on a desk. All data collection was done by group moderators/interviewers AL and NZ. Transcriptions were read several times by the authors and formed the basis for the content analysis. The data were coded to provide an overall description of the issues and to identify recurring themes. The data from the different sources were then triangulated to determine the factors influencing whether pupils participated in the mass-treatment. The quotes are presented to give voice to the participants’ views and indicate whether the respondents were from schools with high or low pupil participation rates in the mass-treatment campaign.

## Results

Six schools participated in this study; in each of these schools two focus group discussions were held for male and female separately, 12 FGD, 75 pupils in all. In addition one female learner, one male learner and one teacher were interviewed individually at these schools. Three traditional healers, one female and two male and a female community health worker were also interviewed

### Perceptions of susceptibility to schistosomiasis

Participants purported that going to the river for laundry, was perceived as a sign of poverty. One of the pupils however, said that parents are very careful how they use the tap water; bathing using tap water was considered as a waste of water, whereas the river has unlimited amounts. Doing laundry in the river also has a sociable aspect, providing a break from the household. Another girl confirmed that rivers are still very much used in daily life: “We collect water from them, drink from them - everything we do from them”.

All participants appeared to know that rivers and dams were places where one might get schistosomiasis. Some said that their parents had been very strict and forbade their swimming and playing in the rivers. Others however told of parents who encourage children to play in rivers. An adolescent pupil said that having schistosomiasis might be perceived as a rural disease, thus being an embarrassment to pupils who then appeared unsophisticated. But rivers flow through all parts of Ugu District, including the peri-urban areas, and many pupils cross rivers on their way to school. In peri-urban and rural areas of Ugu District, many of the bridges are flooded after heavy rains. Pupils are therefore sometimes forced to have water contact on their way to school.

### Perceived benefits of treatment for schistosomiasis

Most participants did not know the benefits of the treatment for schistosomiasis and one third of the informants thought that schistosomiasis is a disease that heals on its own, while some thought that it usually does, and a few said that it always does. One teacher reported that he had been ill with schistosomiasis when he was a young boy, but that it had disappeared on its own after some months. Although the majority of participants did not consider schistosomiasis to be chronic or severe, some had heard that it could cause infertility.

### Sexual association with schistosomiasis

Among the participants, both adults and pupils believed that schistosomiasis could be sexually transmitted. A 17 year old girl said:

“Okay, I think that you can get schistosomiasis sexually because it is something inside you. It’s the blood that is dirty inside you, right? So, when you have intercourse so definitely you will get it because the dirt will come inside you”

Informant 70, School A

One male pupil did not perceive schistosomiasis as a sexually transmitted disease, but was worried that his mother did. He would therefore be very hesitant telling his mother if he had schistosomiasis. According to one of the traditional healers, it depends on age. It appears to be more common to suspect sexual transmission in adults than in children. Generally, schistosomiasis is perceived as a disease that mainly infects children, as they go swimming in lakes and rivers on warm summer days. However, if an adult woman reports that she has schistosomiasis; people may believe that she is using schistosomiasis as a cover for a sexually transmitted infection.

“Many don’t think that you get it by dirty water only; they will say that you are sexually active. That’s why many people don’t want to admit that they have schistosomiasis or even treat it. Because also the nurses at the clinic* - they will first ask you why you have schistosomiasis. They ask you things that are none of their businesses.”

Informant 72 (female), School A

*refers to clinic of primary health care services

A 20 year old male pupil claimed that girls perceive schistosomiasis as sex-related, whereas boys do not. Some pupils believed that the sexual perception of schistosomiasis is gender specific and one teenager said that boys would not mind having sex with a girl even though one of them had schistosomiasis. But according to male informants, girls would not allow it.

### Being teased - a barrier to treatment

When the pupils were asked if they ever talked about schistosomiasis in the schoolyard or among friends, they tended to laugh as schistosomiasis is rarely or never discussed amongst pupils.

When discussing the mass-treatment campaign, pupils were asked about reactions or sanctions from peer pupils when receiving treatment. Some pupils said that if another pupil accepts treatment, the chances are that s/he will be teased by fellow-pupils. They usually did not know why, but said it was fairly common. If a pupil takes the tablet, s/he sends a signal that s/he suspects that s/he has schistosomiasis. Some pupils reported that the praziquantel tablets were occasionally called “dog pills” among pupils, referring to the size of the pill. The term was used to make fun of pupils who had taken them. One 16 year old male pupil spoke of his own experiences from the DoH Mass-treatment Campaign:

“[…] people said many things - that people are making us eat [pills] that are for dogs because they are big. They said they are tablets to treat dogs that we are going to eat. But because I had schistosomiasis, I liked to get help, so I took them.”

Informant 63, School B

During the interview the pupil was asked whether those who chose to take the tablets were laughed at by peer-pupils, he confirmed:

“Yes they laugh, they say… Maybe at the time you are taking the tablets they peep through the windows and say you are getting treated for HIV/AIDS.”

Informant 63, School B

The same boy further reported that his relationship with his peer pupils had changed since he took the schistosomiasis treatment and he spent less time with them.

One female pupil said that she herself had accepted treatment during the DoH Mass-treatment Campaign. She found it hard to take the tablet, as there was a lot of teasing from fellow classmates:

“They said […] that the government wants to decrease the number of people in this world”

Informant 49, School C.

The teasing seemed to cease however, when the disease was cured. One pupil was teased about having schistosomiasis, but as soon as she was well, she herself participated in the teasing of others with schistosomiasis.

### Schistosomiasis as a gender-specific disease

When speaking to the pupils, it was evident that some think schistosomiasis is more common or more damaging to a particular gender. All pupils were asked whether more boys or girls accepted treatment during the DoH Mass-treatment Campaign. A male pupil said that there was a higher participation rate amongst boys than girls:

“Because schistosomiasis is more common in boys, that is why they drink the tablets. And the girls they don't often get schistosomiasis”.

Informant 57, School C

Another boy compared schistosomiasis to women with their menstrual period;

“I think that is the same thing”.

Informant 35, School D.

One of the boys explained why it was more important for boys than girls to take treatment:

“We, boys love to go and swim there yet they [girls] don’t swim. They [girls] plan their stuff but we are unable to do so.”

Informant 4, School F

A 16 year old girl said that in her school, many pupils had the opposite idea:

“Many pupils here at school say that schistosomiasis is in girls and that boys don’t have it.”

Informant 9, School F

Pupils indicated that there were differences between the sexes in terms of participation in the DoH Mass-Treatment Campaign. According to some of the female participants however, there were fewer boys accepting treatment in their school, than girls. After some discussion, they agreed that it is more difficult for boys because of peer pressure. This female focus group (School E, low compliance) also claimed that boys would make more fun of each other than girls, and therefore feared participation in the treatment more.

One of the perceptions concerning schistosomiasis and gender is related to the physical differences between boys and girls. It is sometimes believed to be more serious for girls, as the disease is then internal.

### Fear of side effects

The participants claimed that many pupils did not want schistosomiasis treatment due to the fear of side effects. The participants had not experienced it, but said that they had heard of side effects in nearby schools. When the Department of Health then comes to their schools, pupils fear the treatment, and the participation rate is low. In an individual interview, a girl told us she had refused to take the praziquantel out of fear. When asked what she was afraid of, she said,

“I was scared because other children had taken them and they said these tablets treated them badly in their stomachs.”

Informant 15, School E

These pupils, she said, were from another school in the area

### Lack of trust- a barrier to treatment

**Fig. 2:**
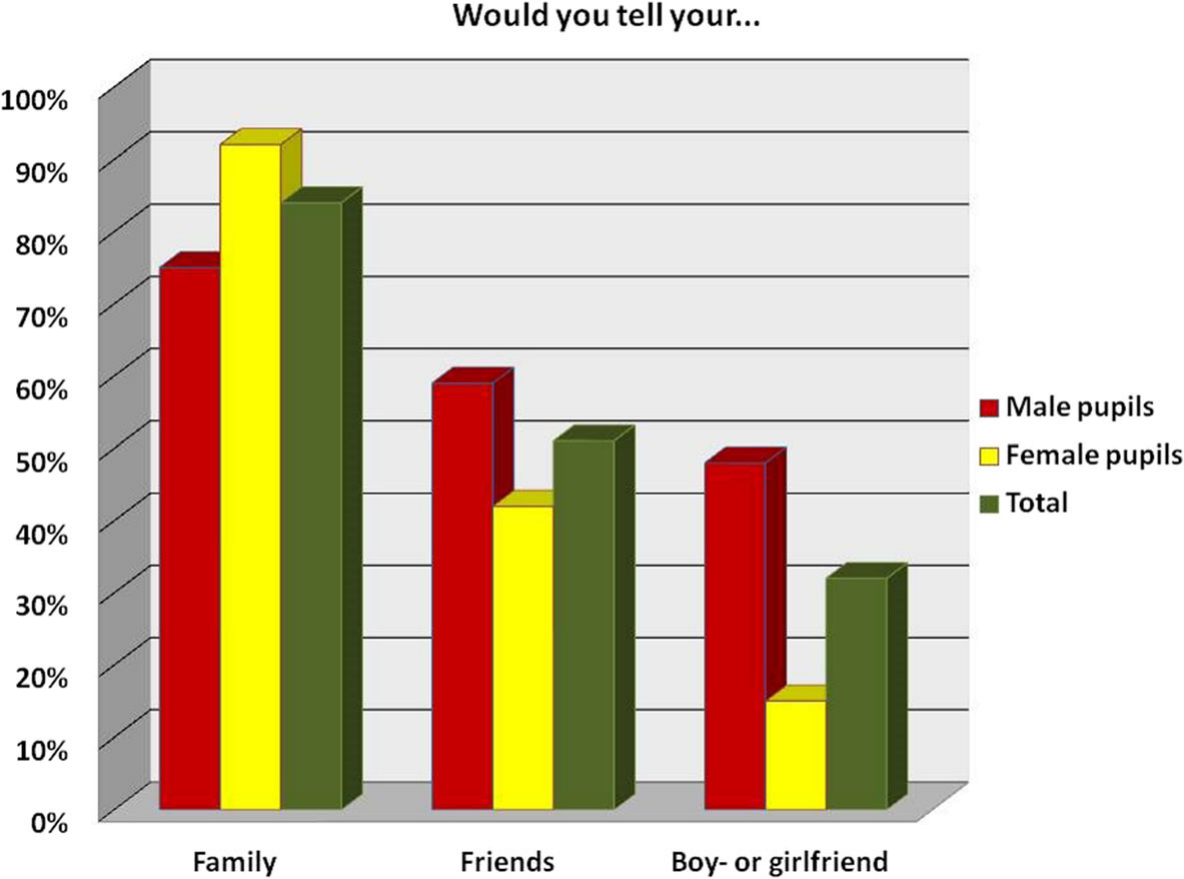
Who would you tell if you had schistosomiasis

The pupils were also asked whom they would be comfortable confiding in if they found that they had schistosomiasis. The participants then discussed amongst themselves whether or not they would tell predefined categories of people. After some discussion, participants who would tell the presented categories were asked to raise their hand, and the numbers are presented in Fig. 2.

Several pupils raised concern about trust although 84% of the interviewed pupils said that they would tell a family member if they had schistosomiasis. Some said they would rather tell a brother or sister than a parent, while others would prefer to tell a grandmother. Most pupils however would still prefer to tell their mother, especially the female participants.

“It’s very hard to tell your parents that’s why I said that I would tell a friend because parents most of the time don’t feel comfortable talking about things that concern your private parts. When you bring it up they just say you are naughty now. So now, if I have schistosomiasis and I am old, then it’s just an embarrassment and parents like talking - they would just tell the entire family.” -

Informant 72, School A

Other participants told of mothers who had hit the child when they found out that their child had schistosomiasis. One 21 year old male pupil had been beaten by his mother to discourage him from swimming in the river again, and another pupil said he was beaten for being naughty in a similar episode.

Pupils said that even if you consider someone your best friend, you cannot be sure that you are their only best friend. When a female focus group was asked whether they would be comfortable telling a boyfriend that they had schistosomiasis, one of the participants replied that she would never tell him.

“I cannot trust people even if we have been in a relationship for seven years, I don’t think I can. Maybe if we were married.”

Informant 12, School E

As shown in Fig. 2, only 30% of participants who responded would want to tell their boy- or girlfriend if they had schistosomiasis.

A few pupils also stressed a lack of trust in doctors and nurses. One female pupil said she feared to go to the local primary health care clinic to get treated for schistosomiasis, because the primary health care nurses would make fun of her. Several others reiterated that statement, saying nurses are not sensitive enough with their patients, and that they sometimes openly make fun of their patients.

### Alternative/traditional treatment

Initially, the participants were asked whether or not schistosomiasis should be treated, and if so, how it should be treated, and were asked to talk about what they knew about schistosomiasis treatment. Everyone knew of the tablets provided through the Department of Health, but most also knew of alternative treatments.

According to the pupils, one has to go to such traditional healers if directed to do so by an elder. Most pupils said however, that they would prefer to go straight to a clinic of modern medicine.

### Misconceptions

A misconception presented by pupils as well as teachers is the belief that schistosomiasis is not a current problem. A female pupil said that people from her community just do not know enough about schistosomiasis;

“They tell themselves that it is a disease that passed long time ago. Also even if they hear about it they take it lightly because they have not heard that anyone died of schistosomiasis”.

Informant 40, School D

### Need for more information

Several of the adult participants (teachers and community participants) said that the transfer of knowledge between parents and their children was more common 40 years ago than currently. When asked why, they argued that the general communication between parents and their children had changed for several reasons. Many children do not live with their parents.

Study participants observed that children no longer talk to their parents about their everyday challenges. According to these participants, one would only communicate with parents when being reprimanded. This may not be an accurate description of all households, but many pupils confirmed that they do not speak to either of their parents about everyday challenges. According to the adult participants, parents used to teach their children about common health conditions, challenges and changes previously. In some families, it was considered the elder’s responsibility to educate the grandchildren. However, most participants did not have grandparents, as they had passed away.

“People in the community have that thing that you get schistosomiasis if you want to go and swim. They shout at children when they are young that they should not go and swim since they will have schistosomiasis. They have never just sat down and talked to us and told us that this is schistosomiasis and this is what it causes. They have never - you grow up and find it out yourself”.

Informant 7, School A

This 17 year old girl describes an authoritative communication; simply being told that one should not swim due to schistosomiasis might in many cases not be sufficient information.

According to the pupils schistosomiasis had not been addressed in any of the school subjects. On one occasion, a female focus group participant said she believed some highlights were mentioned in the school subject “Life Orientation” [24]. Teachers said it could be incorporated into the Life Orientation syllabus, but felt that the curriculum is already cluttered with topics. One teacher said that schistosomiasis should absolutely be included in their teaching material. When he was asked to explain why, he responded:

“It should be there, why, you see, these things we are talking about – people’s health. We’re talking about people’s futures. They should be empowered, they should know”.

Teacher Informant 29.

The majority of the participants said that their knowledge about schistosomiasis had been acquired during the DoH Mass-Treatment Campaign.

## Discussion

Pupils were often teased about having schistosomiasis. Some also reported that nurses would make fun of them if they visited the primary health care clinic, intending to test for schistosomiasis. Pupils claimed that the main reason for non-participation probably is the fear of being teased by peers. Although we have reported on a local problem of low participation in mass-treatment another study in Uganda amongst adults has reported similar constraints in achieving the required 75% target coverage [25]. Perceptions about poverty, sexually transmitted infections, and being dirty are notions that may cause serious social damage to a pupil and may lead to stigma [26]. The risk of teasing greatly adds to the dimension of “perceived barriers” as presented in the Health Belief Model [23]. Furthermore, some pupils claimed to lose contact with friends as a consequence of having schistosomiasis. In many discussions and interviews schistosomiasis was mentioned as an embarrassment. Pupils who had refused treatment said that they would have taken the treatment in private. However, others disagreed and said that they would prefer to take it in the classroom, with peers present.

Stigma related to a health issue or disease may be defined as “social disqualification of individuals and populations who are identified with particular health problems” [27]. More specifically, a person who is a victim of health-related stigma experiences exclusion, judgment and rejection based on the health situation. The stigma cannot be justified based on medical grounds, but is a cultural phenomenon [28]. Such exclusion will further affect a person’s social life, challenging and reducing their ability to prosper [29]. Further, a person’s access to health care may decline if s/he is/or perceives her/himself to be a target of stigmatization through fear of being seen seeking treatment. [28, 30].

Schistosomiasis has previously been suggested to carry with it a social burden of stigma, especially urinary schistosomiasis [31, 32]. Furthermore; several participants raised concern about the sexual transmission of schistosomiasis, an incorrect perception that may increase the possibility of stigma [26]. In addition there are also physical consequences resulting from schistosomiasis such as infertility that could cause stigma [8].

### Perceptions of susceptibility to schistosomiasis

Laundry, bathing and swimming takes place in rivers, lakes and dams. As late as year 2001 33% of the inhabitants of Ugu did not have any access to piped water [33]. As access to clean water has increased [34], many households now have the facilities to do laundry and bathing nearer to home.

### Perceived benefits of treatment for schistosomiasis

Fertility is important in Zulu culture, and a woman’s value has been measured by her ability to produce babies [35]. A proved association with infertility might be a motivational factor for pupils to accept treatment.

The study population, however, considered schistosomiasis’ impact to be minor. According to the Health Belief Model, perceptions of low severity would decrease the will to participate in a treatment campaign. To improve participation it is therefore of great importance that measures are taken to increase the knowledge about the potential schistosomiasis-related consequences. Teachers may have an important role in explaining the rationale for treatment and to encourage pupils to participate. One pupil said that fear of infertility motivated her to accept treatment, a cue to action in terms of the Health Belief Model [23].

### Sexual association with schistosomiasis

The modes of transmission for schistosomiasis (through water) and sexually transmitted diseases are completely different [36, 37], but *S. haematobium* (urogenital schistosomiasis) causes symptoms that are also typical of sexually transmitted diseases [38, 39]. This has been well documented in females but is largely unexplored in males [40–43]. This may be why participants associate schistosomiasis with sexually transmitted diseases. This study did not show that perceptions of schistosomiasis are limited to females in influencing participation rates in the Ugu mass-treatment campaign. The pupils acknowledge that both male and female are susceptible to schistosomiasis, although many lack adequate information about the disease.

### Fear of side effects

Side-effects are thought to be a reaction to the death of the worms, and most side-effects observed in school treatment programmes occur during the first round of treatment; when children harbour a large worm load [19]. Mild abdominal pain, nausea, vomiting, diarrhoea and fatigue are the most frequently reported adverse effects, and the recipients do not require medical attention. There had been information visits to the schools prior to the treatment days [22]. There are two major benefits of anti-schistosomal treatment, as perceived by participants, namely alleviating the symptoms such as blood in the urine and dysuria, and the prevention of potential consequences such as infertility. Pupils must understand the positive effects of treatment, as a counterpoint to the risk of side effects. The fact that pupils are provided with food prior to treatment in order to reduce the possibility of side-effects must also to be explained to potential participants. The fear of side effects should not have a large effect on participation rates, based on scientific reasoning. If they understand the benefits of treatment and the need for a cure for schistosomiasis, they may endure the risk of side effects which are rarely serious. In addition to the risk of being teased, the fear of side effects is a powerful contributor to the “perceived barriers” constituting a cofactor in non-participation in the mass-treatment campaign. It is therefore evident that by reducing the fear of side effects through health education, participation rates could feasibly increase.

### Lack of trust- a barrier to treatment

Lack of trust in the health personnel may plausibly affect the trust of the DoH school nurses who come to the schools during the mass-treatment campaign. If the general perception of health workers is that they cannot be trusted, and this it may contribute to the lack of confidence in the treatment. Lack of trust in friends, family, romantic partners and also the authorities were recurring concerns throughout the pupil interviews. The pupils did not mention this as a reason for non-participation, but as a reason for not talking openly about schistosomiasis, whether infected or not.

### Alternative/traditional treatment

In Zulu tradition, there are different types of illnesses, categorized by beliefs in the cause of the illness. Briefly, the two major groups are “ukufa kwabantu” and “umkhuhlane”, where the first category consists of illnesses specific to African people, and hence the need for African remedies [44]. The latter, “umkhuhlane” consists of diseases that simply occur in all societies, and that can, if treatable, be treated by modern medicine [45]. Schistosomiasis was categorized as the latter according to some of the study participants. However, other pupils reported that they were referred to traditional healers and herbalists by parents or elderly relatives for the treatment of schistosomiasis [46].

### Misconceptions

Schistosomiasis is considered the second most devastating parasitic disease globally [47] in terms of public health impact. Schistosomiasis causes additional challenges in poor people’s fight to break out of poverty [9, 14, 20]. Mortality rates for schistosomiasis have probably been underestimated, and it is believed that 200,000 people die every year in Sub-Saharan Africa from the chronic consequences of schistosomiasis [21]. The data implied that a wide range of misconceptions concerning schistosomiasis are evident in Ugu District, the perception of sexual association may be the most common one. This perception can also be severely damaging, as it may lead to false accusations such as assuming virgins are sexually active or that you or your partner have been unfaithful. Firstly however, the inhabitants of Ugu District must be made aware that urogenital schistosomiasis is a serious problem in their district. Pupils need to understand the potential consequences of schistosomiasis. As long as they do not believe schistosomiasis is a current problem, and believe that it may do little harm, there is limited support for mass-treatment and it will be difficult to combat the disease. Basic knowledge about schistosomiasis must be provided before further measures in fighting the disease can be undertaken [2]. Misconceptions that may lead to teasing, general knowledge about schistosomiasis may thus wane. Meetings in the community could be arranged for all age groups, including traditional healers, local health workers, elderly, parents and children. In line with the Health Belief Model, all of the perceptions need to be challenged to increase participation in Mass-Treatment to reach the required 75% treatment coverage [21]. More information may also encourage the traditional healers to refer patients to health clinics for treatment.

### Need for more information

Some pupils stay with a guardian or a more distant relative, and as a result of the AIDS epidemic some children live in child-headed households [48]. The mode of health communication may therefore be interrupted as guardians may not feel the same obligation to educate a child as would a parent. Therefore education in schools is necessary.

Previous research shows that Mass-Treatment Campaigns are not successful unless transmission is reduced/interrupted [49]. The lack of knowledge or poor information about schistosomiasis may constitute a breeding-ground for misconceptions that may detrimentally influence participation and hesitance toward treatment by adding to the root of teasing. In order to achieve the necessary reduction in prevalence, knowledge must be increased in Ugu District, enabling the Ugu population to take the necessary precautions to avoid re-infection.

Through increased knowledge, pupils in Ugu high schools will understand that urogenital schistosomiasis is a serious disease and that it needs to be treated with praziquantel. By clarifying the perceived association between urogenital schistosomiasis and sexual behaviour, the grounds for teasing will also be reduced. If the benefits and relief provided by treatment are understood, these might overshadow the fear of side effects. This knowledge combined with free treatment through the Department of Health’s Mass-treatment Campaign may be sufficient cues for action. All dimensions of the Health Belief Model will be positively affected, and encourage participation.

## Conclusion

As long as schistosomiasis is believed to be harmless, eradicated or of low severity, there will be a lack of motivation for actions to reduce the infection. The Health Belief Model indicates that with increased knowledge, perceptions about the severity and susceptibility to schistosomiasis and with cues to action for treatment, participation rates for the mass-treatment campaign may increase [23], thus decreasing the schistosomiasis prevalence in Ugu District. More attention is needed to addressing the population’s concerns in order to facilitate increased uptake of praziquantel, and to reduce the deleterious effects of schistosomiasis, which appears to be associated with increased risk of HIV. Infrastructure such as bridges, protected water, and sanitation must to be improved in rural areas in order to decrease infection [50, 51]. A well-planned and structured information campaign in several neighbouring schools should be organized before treatment is given. Further studies are needed to explore if adolescent participation would increase if treatment is offered in private rooms.

## Abbreviations

AIDS: Acquired Immunodeficiency Syndrome
Clinic: Refers to clinic of primary health care services
DoH: Department of Health
FGD: Focus Group Discussions
HBM: Health Belief Model
HIV: Human Immunodeficiency Virus
KZN: KwaZulu-Natal
WHO: World Health Organisation
